# Expansion in CD39^+^ CD4^+^ Immunoregulatory T Cells and Rarity of Th17 Cells in HTLV-1 Infected Patients Is Associated with Neurological Complications

**DOI:** 10.1371/journal.pntd.0002028

**Published:** 2013-02-07

**Authors:** Fabio E. Leal, Lishomwa C. Ndhlovu, Aaron M. Hasenkrug, Fernanda R. Bruno, Karina I. Carvalho, Harry Wynn-Williams, Walter K. Neto, Sabri S. Sanabani, Aluisio C. Segurado, Douglas F. Nixon, Esper G. Kallas

**Affiliations:** 1 The Division of Experimental Medicine, Department of Medicine, University of California San Francisco, San Francisco, California, United States of America; 2 Hawaii Center of AIDS, Department of Tropical Medicine, John A. Burns School of Medicine, University of Hawaii, Honolulu, Hawaii, United States of America; 3 Deparment of Infectious Diseases, School of Medicine, University of Sao Paulo, Sao Paulo, Brazil; 4 Division of Clinical Immunology and Allergy, University of Sao Paulo Medical School, Sao Paulo, Brazil; 5 Molecular Biology Laboratory, Fundação Pró-Sangue, Hemocentro de São Paulo, Brazil; 6 Department of Translational Medicine, Federal University of São Paulo, São Paulo, Brazil; George Mason University, United States of America

## Abstract

HTLV-1 infection is associated with several inflammatory disorders, including the neurodegenerative condition HTLV-1-associated myelopathy/tropical spastic paraparesis (HAM/TSP). It is unclear why a minority of infected subjects develops HAM/TSP. CD4^+^ T cells are the main target of infection and play a pivotal role in regulating immunity to HTLV and are hypothesized to participate in the pathogenesis of HAM/TSP. The CD39 ectonucleotidase receptor is expressed on CD4^+^ T cells and based on co-expression with CD25, marks T cells with distinct regulatory (CD39^+^CD25^+^) and effector (CD39^+^CD25^−^) function. Here, we investigated the expression of CD39 on CD4^+^ T cells from a cohort of HAM/TSP patients, HTLV-1 asymptomatic carriers (AC), and matched uninfected controls. The frequency of CD39^+^ CD4^+^ T cells was increased in HTLV-1 infected patients, regardless of clinical status. More importantly, the proportion of the immunostimulatory CD39^+^CD25^−^ CD4^+^ T-cell subset was significantly elevated in HAM/TSP patients as compared to AC and phenotypically had lower levels of the immunoinhibitory receptor, PD-1. We saw no difference in the frequency of CD39^+^CD25^+^ regulatory (Treg) cells between AC and HAM/TSP patients. However, these cells transition from being anergic to displaying a polyfunctional cytokine response following HTLV-1 infection. CD39^−^CD25^+^ T cell subsets predominantly secreted the inflammatory cytokine IL-17. We found that HAM/TSP patients had significantly fewer numbers of IL-17 secreting CD4^+^ T cells compared to uninfected controls. Taken together, we show that the expression of CD39 is upregulated on CD4^+^ T cells HAM/TSP patients. This upregulation may play a role in the development of the proinflammatory milieu through pathways both distinct and separate among the different CD39 T cell subsets. CD39 upregulation may therefore serve as a surrogate diagnostic marker of progression and could potentially be a target for interventions to reduce the development of HAM/TSP.

## Author Summary

Human T-lymphotropic virus type 1 (HTLV-1) has been estimated to infect 10–20 million worldwide. The majority of infected individuals are asymptomatic, however, 2% to 3% develop a neurodegenerative disorder called HTLV-1-associated myelopathy/tropical spastic paraparesis (HAM/TSP). The reasons why persons with HTLV-1 develop these complications appear to be multiple and complex. Cellular immune response has been implicated in the development of inflammatory alterations in these patients, however the pathogenic mechanisms for disease progression remain unclear. Regulatory CD4^+^ T cells (Treg) and Th17 cells derive from a common progenitor and conflicting results regarding frequency and function are found in the development of HAM/TSP. The expression of the CD39 ectoenzyme, a molecule that can mediate immunostimulatory and inhibitory effects, is useful to define IL-17 secreting cell populations, suppressive CD4^+^ T cells and CD4^+^ T cells with immunostimulatory properties. The interplay of these T-cell subsets may reveal important aspects of HAM/TSP pathogenesis. In this study, we performed an evaluation of the immunoregulatory CD4^+^ T-cell subsets defined by CD39 expression including Th17 cells. Our results present phenotypic and functional alterations in the CD4^+^ T cell profile that could account for the transition from asymptomatic status to HAM/TSP, predicting clinical disease risk and tracking disease progression.

## Introduction

Human T-lymphotropic virus type 1 (HTLV-1) has been estimated to infect 10–20 million worldwide [1]. The majority of infected individuals remain asymptomatic carriers of this retrovirus for life. However, 2% to 3% of HTLV-1-infected individuals develop a neurodegenerative disorder characterized by a progressive spastic paraparesis called HTLV-1-Associated Myelopathy/Tropical Spastic Paraparesis (HAM/TSP) [2], [3]. Other chronic inflammatory conditions including arthritis, uveitis, polymyositis, and Sjögren syndrome have also been associated with HTLV-1 infection [4], [5], [6], [7]. In endemic areas, 2% to 6% of seropositive individuals develop Adult T-cell Leukemia (ATL) [8]. In the absence of efficient treatment options that modify disease progression and protective vaccination, understanding the causative mechanisms of disease progression is paramount to develop preventative and treatment options.

The reasons why persons with HTLV-1 infection develop these complications appear to be multiple and complex, and the mechanisms for progression have not been fully determined. Several mechanisms have been postulated to account for disease progression to HAM/TSP such as age, gender, transmission mode and proviral load levels [9], [10], [11], [12], [13]. Cellular immune response has been implicated in the control of HTLV-1 infection as well as in the development of inflammatory alterations in these patients. The viral protein Tax is the immunodominant peptide recognized by CD8^+^ T cells in patients with HTLV-1. Analyses of the role of HTLV-1 Tax-specific CD8^+^ T cells in the control of HTLV-1 infection show that strong CD8^+^ T cytolytic activity correlates negatively with proviral load, but it occurs regardless of disease status [14], [15]. The higher frequencies of HTLV-1-Tax-specific IFN-γ^+^ CD8^+^ T cells are positively associated with the frequency of HTLV-1-infected cells in HAM/TSP patients suggesting that CD8^+^ T cell responses may neither control viral replication nor prevent disease progression [16], [17]. Such high frequency of HTLV-1-Tax-specific IFN-γ-producing CD8^+^ T cells, with low expression of inhibitory receptors in peripheral blood and in the central nervous system appear to contribute to the inflammatory alterations seen in HAM/TSP patients [18], [19], [20], [21].

Recently, the viral protein HTLV-1 basic leucine zipper (HBZ), encoded by an anti-sense strand of the HTLV-1 provirus [22], [23], may better serve as proxy for disease progression than Tax. HBZ expression down-regulates Tax expression [23], inhibit NF-κB classical pathway activation and yet promotes CD4^+^ T-cell proliferation in transgenic mice [24], [25]. HBZ-specific CD8^+^ T cells, though not as frequent as Tax-specific CD8^+^ T cells, appear to correlate with proviral load and disease progression, represents a potential target for HAM/TSP progression and provide important clues for disease progression [26], [27]. These studies, however, may only partially account for the transition from an asymptomatic status to the development of HAM/TSP.

HTLV-1 infects several human cell types [28], [29], but primarily CD4^+^ T cells [30], [31]. Under specific conditions, CD4^+^ T cells differentiate towards Th1, Th2, Treg and Th17 lineages [32], [33], [34]. Distinct CD4^+^ T-cell subsets play a pivotal role on the immune response. Regulation of HTLV-1 infection and CD4^+^ T-cell subsets frequency and function may be influenced by the expression of viral proteins Tax and HBZ that activates promoters of several cellular genes and induces CD4^+^ T cell replication [25]. Furthermore, *HBZ* transcription has been reported to correlate with proviral load, inflammatory markers and disease severity [27], [35]. Higher frequencies of virus-specific IFN-γ-producing CD4^+^ T cells are observed in cerebrospinal fluid (CSF) and sera of HAM/TSP patients compared to HTLV-1 asymptomatic carriers with similar proviral load [36], suggesting a role for CD4^+^ T cells in neural damage. Understanding immune regulatory aspects and CD4^+^ T-cell responses to HTLV-1 could clarify the complex pathogenesis of HAM/TSP in the midst of strong anti-HTLV-1 immunity. Specific CD4^+^ T-cell subsets play a key role in the regulation of immune responses and inflammatory diseases [37], [38] and modulate the function of CD8^+^ T cells, including Tax-specific CD8^+^ T cells cytolytic activity [39]. Two antagonistic subsets involved in the pathway of tolerance and immunity, regulatory CD4^+^ T cells (Treg) and Th17 cells, derives from a common progenitor [40] and conflicting results regarding frequency and function are found in studies of Tregs and Th17 in the development of HAM/TSP [39], [41], [42], [43], [44], [45].

The CD39 ectoenzyme can mediate immunostimulatory and inhibitory effects by releasing adenosine through its enzymatic activity [46]. Our previous study and those by others have shown that expression of CD39 serves as a novel marker to identify suppressive CD4^+^ T cells [47], a CD4^+^ T-cell subset with immunostimulatory properties [48] and can further distinguish between IL-17 secreting cell populations [49]. The interplay of these T-cell subsets may reveal important aspects of HAM/TSP pathogenesis. Furthermore, depletion of Th17 cells in the peripheral blood and in the gut is detrimental to control of HIV-1 infection, another retrovirus with many similarities to HTLV-1 acquisition but with divergent clinical outcomes [50], [51], [52]. It is unclear whether Th17 cells may contribute to the immune response to HTLV-1 replication as well as to the proinflammatory milieu seen in HAM/TSP patients.

In this study we performed an evaluation of the immunoregulatory CD4^+^ T-cell subsets defined by CD39 expression including Th17 cells in patients enrolled in an HTLV-1 clinic in Sao Paulo, Brazil. We hypothesized that changes in the CD4^+^ T cell compartment would lead to alterations in T-cell functions that may be involved in HTLV-1 disease progression. Our results present phenotypic and functional alterations in the CD4^+^ T-cell profile based on CD39 expression that could account for the transition from an asymptomatic status to HAM/TSP, predicting clinical disease risk and possibly track disease progression.

## Materials and Methods

### Ethics Statement

All human participants of this study voluntarily signed an informed consent approved by the institutional review board of the University of Sao Paulo (IRB #0855/08) Sao Paulo, Brazil. Clinical investigation procedures were conducted according to the principles expressed in the Declaration of Helsinki (http://www.wma.net/en/30publications/10policies/b3/index.html)

### Study Participants

We enrolled 37 patients from the HTLV-1 Outpatient Clinic at the University of Sao Paulo, Brazil. They were invited to participate in a longitudinal cohort of HTLV-1-infected subjects after signing a written informed consent approved by the University of Sao Paulo's Institutional Review Board (#0855/08). This cohort includes 24 asymptomatic carriers (AC) and 13 patients with neurological complications related to HTLV-1 infection denominated HTLV-1 Associated Myelopathy/Tropical Spastic Paraparesis (HAM/TSP). The clinical status was determined based on WHO criteria for HTLV-1 associated diseases [53]. The majority of the patients were female (67%), 17 in the asymptomatic group and 8 HAM/TSP patients, with a median age of 47 (Interquartile Range [IQR], 36–55) and 54 (IQR, 36–61) years respectively.

We enrolled 19 age- and gender-matched healthy volunteers without laboratory evidence of HTLV-1, Hepatitis B, Hepatitis C, and HIV infections, with similar demographic characteristics as the HTLV-1-infected participants (See Table 1).

**Table 1:** Characteristics of study participants.

	Uninfected (n = 19)	Asymptomatic carrier (n = 24)	HAM/TSP† (n = 13)	P value
Age, median (IQR‡)	39 (29–52)	47 (36–55)	54 (36–61)	NS
Gender (male/female)	6/13	7/17	5/8	
CD4+ T cells, per ml (mean ± SD**)	1217±419.4	1152±432.3	1305±606.6	NS
CD4+ T cell percentage (mean ± SD)	48.75±7.16	54.6±10.92	54.54±7.80	NS
HTLV-1* proviral load, copies/10^3^ cells (mean ± SD)	N/A	68.91±124.61	249.38±302.42	0.0026

† HAM/TSP: HTLV-1 associated myelopathy/tropical spastic paraparesis;

* HTLV-1: Human T Lymphotropic Virus Type 1;

‡ IQR: Interquartile Range, 25%–75%;

** Standard Deviation.

Blood samples were obtained and processed with Ficoll-Paque PLUS (Amersham Pharmacia Biotech, Uppsala, Sweden) gradient centrifugation and peripheral-blood mononuclear cells (PBMC) were isolated and cryopreserved in 10% DMSO in FBS. This study was approved by the institutional review board and ethical committee of the University of Sao Paulo (#0855/08).

### Flow Cytometry Assessment

Cryopreserved PBMC were thawed in RPMI 1640 with 10% FBS and washed in FACS buffer (PBS with 0.5% bovine serum albumin, 2 mM EDTA). Phenotypic detection was performed on 10^6^ cells by incubation with conjugated anti-CD3, CD4, CD25, CCR4 (BD Biosciences, San Diego, CA), PD-1 (Biolegend, San Diego, CA) and CD39 (eBioscience, San Diego, CA) for 30 minutes on ice. For intracellular staining, cells were fixed and permeabilized prior to staining with conjugated antibodies against FoxP3, IFN-γ, IL-10, IL-2 (BD Biosciences), CTLA-4 (Immunotech, Marseille, France), TNF-α (eBioscience) and IL-17 (Biolegend, San Diego, CA).

#### Proviral load and mRNA assessment

HTLV-1 proviral DNA was extracted from PBMCs using a commercial kit (Qiagen GmbH, Hilden Germany) following the manufacturer's instructions. The extracted DNA was used as a template to amplify a fragment of 158 bp from the viral tax region using previously published primers [54]. The SYBR green real-time PCR assay was carried out in 25 µl PCR mixture containing 10× Tris (pH 8.3; Invitrogen, Brazil), 1.5 mM MgCl_2_, 0.2 µM of each primer, 0.2 mM of each dNTPs, SYBR Green (18.75 Units/r×n; Cambrex Bio Science, Rockland, ME) and 1 unit of platinum Taq polymerase (Invitrogen, Brazil). The amplification was performed in the Bio-Rad iCycler iQ system using an initial denaturation step at 95°C for 2 minutes, followed by 50 cycles of 95°C for 30 seconds, 57°C for 30 seconds, and 72°C for 30 seconds. The human housekeeping β-globin gene primers GH20 and PC04 [55] were used as an internal control calibrator. For each run, standard curves for the value of HTLV-1 tax were generated from MT-2 cells of log_10_ dilutions (from 10^5^ to 10^0^ copies). The threshold cycle for each clinical sample was calculated by defining the point at which the fluorescence exceeded a threshold limit. Each sample was assayed in duplicate and the mean of the two values was considered as the copy number of the sample. The amount of HTLV-1 proviral load was calculated as follows: copy number of HTLV-1 (tax) per 1,000 cells = (copy number of HTLV-1 tax)/(copy number of β globin/2)×1000 cells. The method could detect 1 copy per 10^3^ PBMC.

For the mRNA quantification assays, RNA was extracted using QIAamp RNA Blood Mini Kit (Qiagen) following the manufacturer's instructions and at a final 50 µl elution. The transcription levels of Tax and HBZ and internal reference β-Actin were measured by The *Power SYBR Green* RNA-to-C_T_ 1-Step kit (Life Technologies, Carlsbad, CA) using the StepOnePlus Real-Time PCR System (Life Technologies). *Tax* and *HBZ* specific primers were used to measure the respective mRNA expression level as described previously [27]. β-actin was used as a housekeeping control to calculate 2^−ΔΔCt^ relative expression as previously described [56]. This method allows measuring the relative expression of each gene to an endogenous control, and normalizes measurements as has been previously shown [57].

### ELISPOT Assays

MAIPS4510 Elispot plates (Millipore, Danvers, MA) were coated with anti-IL-17 or anti-IFN-γ 10 mg/ml (Mabtech, Nacka Strand, Sweden) in PBS, 50 ml/well for one hour at room temperature. After three washes with PBS, PBMC (1×10^5^ cells/well) and Phorbol 12-myristate 12-acetate (PMA) and ionomycin (Ion) (Sigma, St Louis) were added, with a final volume of 200 µl/well. Plates were incubated at 37°C in 5% CO_2_ for 16–20 hours. After washing with phosphate-buffered saline (PBS) plus 0.1% Tween 20 (PBST), biotinylated anti-IL-17 or anti-IFN-γ (1 mg/ml) (Mabtech), antibodies were added to the appropriate wells in PBS 0.1% Tween 1% BSA (PBSTB) for 30 minutes at room temperature. Plates were washed again three times with PBST, and alkaline phosphatase-conjugated streptavidin (Jackson Immunoresearch, West Grove, PA) was added (50 ml of 1∶1,000 dilution in PBSTB) and incubated for 30 min at room temperature. Plates were washed in PBST, incubated with blue substrate (Vector Labs, Burlingame, CA) until spots were clearly visible, then rinsed with tap water. When plates were dry, spots were counted using an automated ELISPOT reader. Experiments run in duplicate for IL-17 and IFN-γ detection. Results were medium-subtracted and normalized to 10^6^ cells. IFN-γ spots were considered positive controls.

### Statistical Analysis

Statistical analysis was performed by using GraphPad Prism statistical software (GraphPad Software, San Diego, CA). Non-parametric statistical tests were used throughout the analyses. The Mann-Whitney U was used for comparison tests and the Spearman rank test were used for correlation analyses.

## Results

### Higher Frequency of CD39 Expressing CD4^+^ T Cells Subsets in HAM/TSP Infection

The evaluation of CD4^+^ T cells subsets as defined by the expression of CD39 and CD25 has revealed novel functional populations that redefine suppressor T cells expressing FoxP3 [47], [58], [59], delineate Th17 cells and identify a population of CD39 expressing T cells with immunostimulatory properties called “inducer” cells [48], [59], [60]. The expression of these markers in HTLV-1 remains undefined.

**Figure 1:**
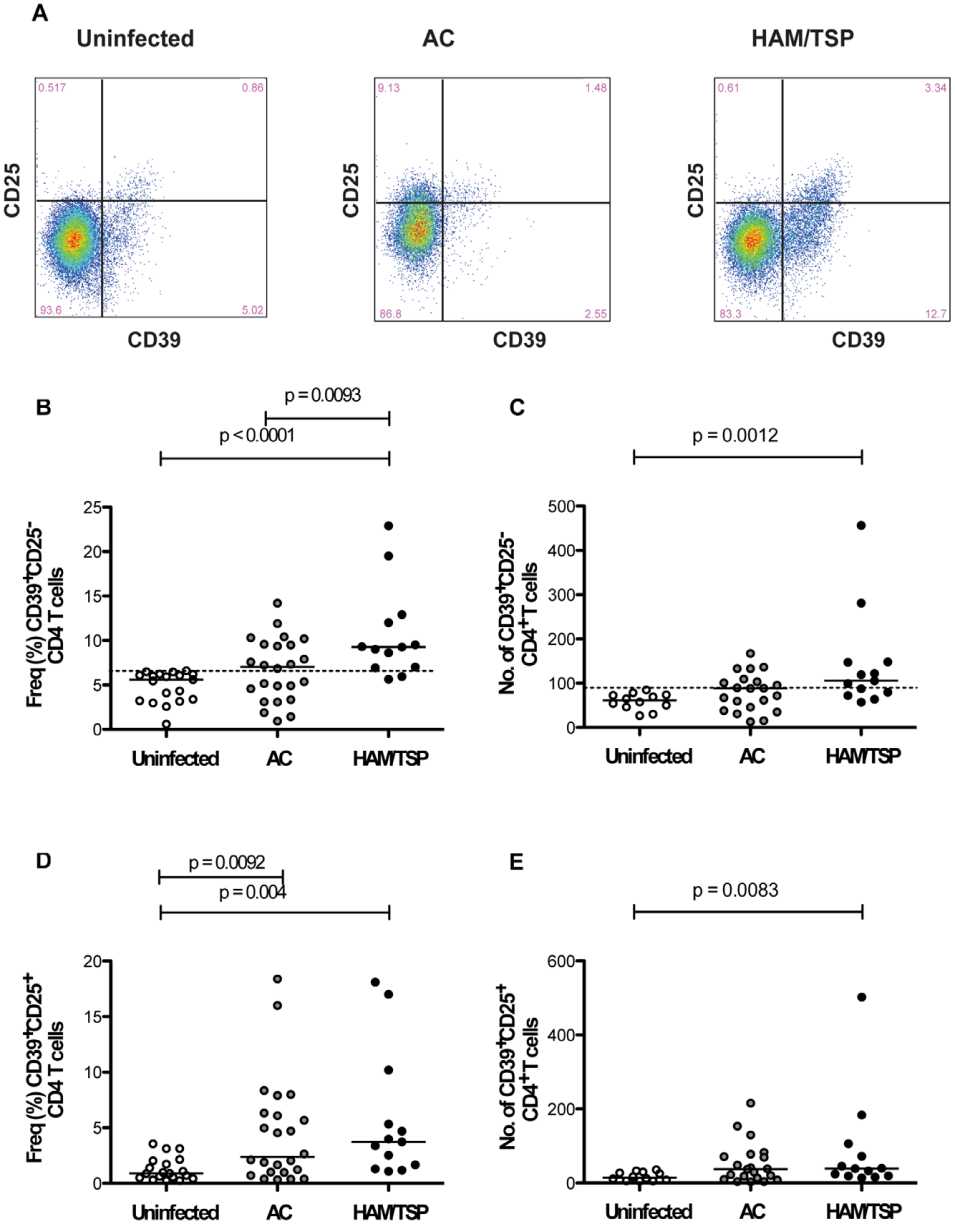
CD25 and CD39 expression in CD4^+^ T cells. Compare results from HAM/TSP patients, HTLV-1 asymptomatic carriers (AC) and uninfected subjects. The statistical difference was deemed significant using a Mann-Whitney U test analysis if p<0.05. Horizontal bars denote median values. (A) CD39 and CD25 expression in CD4^+^ T cells from one representative uninfected donor, one HTLV-1-infected asymptomatic carrier and one HAM/TSP patient. (B) Proportion of CD39^+^CD25^−^CD4^+^ T cells. Dotted line represents higher levels of CD39^+^CD25^−^ CD4^+^ T cells in uninfected subjects. (C) Number of CD39^+^CD25^−^CD4 T cells. (D) Proportion of CD39^+^CD25^+^CD4^+^ T cells. (E) Number of CD39^+^CD25^+^CD4^+^ T cells.

We examined, by flow cytometry, the pattern of expression of CD39 and CD25 in CD4^+^ T cells of 19 uninfected subjects, 24 HTLV-1 asymptomatic carriers and 13 HAM/TSP patients. Since the transcription factor FoxP3 serves to define suppressor CD4^+^ T cells, we first determined its distribution within the CD39^+^ CD4^+^ T cells. Based on our gating strategy and in line with our previous study [48], we confirmed that the distribution of FoxP3 within the CD4^+^ T-cell subsets using CD39 and CD25 (CD39^+^FoxP3^+^CD25^+^ and CD39^+^FoxP3^+^CD25^−^ and CD39^−^FoxP3^+^CD25^+^) did not change irrespective of HTLV-1 infection (data not shown). We observed that the frequencies of CD39^+^CD25^−^CD4^+^ T cells were significantly higher in HAM/TSP compared to AC and uninfected subjects (Fig. 1A,B). Similarly, the numbers of CD39^+^CD25^−^CD4^+^ T cells were significantly higher in HAM/TSP patients compared to uninfected subjects (Fig. 1C). Significant higher frequencies of CD39^+^CD25^+^CD4^+^ T cells were found in AC and HAM/TSP patients compared to uninfected subjects, but the frequencies between the two groups of HTLV-1 infected patients were not significantly different (Fig. 1D). The numbers of CD39^+^CD25^+^CD4^+^ T cells were also significantly increased between HAM/TSP patients compared to uninfected subjects (Fig. 1E). We observed no differences in the absolute CD4^+^ T cell count (Fig. S2A), frequency (Fig. S2B), and number (Fig. S2C) of CD39^−^CD25^+^CD4^+^ T cells between uninfected donors, AC, and HAM/TSP patients. We and others have observed that T regulatory cells can be further defined by the low expression of CD127 and FoxP3 [61], [62]. To further define the proportion of regulatory T cells subsets in HTLV-1 infection, we used a combination of antibodies anti-FoxP3, CD127, and CD25. We determined that phenotypically defined regulatory (CD127loCD25^+^FoxP3^+^) CD4^+^ T cells were also elevated in HTLV-1 infection using this combination of markers. (Fig. S1).

The high frequencies of CD39^+^CD25^+^CD4^+^ T cells in AC and HAM/TSP subjects reinforce the idea of induced Treg differentiation in HTLV-1 infection while the high frequency of CD39^+^CD25^−^ CD4^+^ T cells only in HAM/TSP patients may contribute to the proinflammatory milieu seen in HAM/TSP and represent a marker of disease progression.

### Changes in the Expression of CCR4, CTLA-4 and PD-1 among the CD39 Expression CD4 T Cells in HAM/TSP

**Figure 2:**
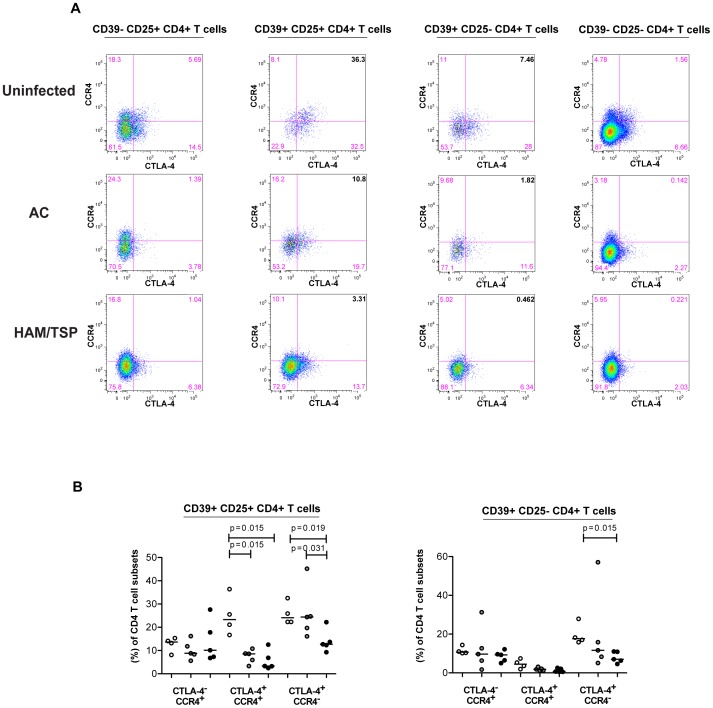
Expression of CTLA-4 and CCR4 in CD4^+^ T-cell subsets based on CD39 and CD25 expression. The statistical difference was deemed significant using a Mann-Whitney U test analysis if p<0.05. Horizontal bars denote median values. (A) CTLA-4 and CCR4 expression on CD4^+^ T cells from one representative uninfected donor, one HTLV-1-infected-asymptomatic carrier and one HAM/TSP patient. (B) Proportion of expression of CTLA-4 and CCR4 in CD39^+^CD25^+^ and CD39^+^CD25^−^ CD4^+^ T cells of uninfected donors, AC and HAM/TSP patients.

CTLA-4 is essential for regulatory T-cell suppressive function as blockade of CTLA-4 expression abrogates Treg function [63], [64]. CCR4, a chemokine receptor selectively expressed on Th2, Th17 and Tregs, has been shown to be highly expressed by HTLV-1-infected cells [65]. Furthermore, CCR4 expressing CD25^+^ CD4^+^ T cells are functionally altered in HAM/TSP patients, producing high levels of IFN-γ [44]. In an effort to further characterize the two immunoregulatory T cell populations defined by CD39 and CD25 expression, we evaluated the expression of CTLA, CCR4 and PD-1 among these subsets. We observed that CD39^+^CD25^−^ CD4^+^ T cells had the highest expression of CCR4 whereas the CD25^+^CD39^+^ CD4^+^ T cells had greatest co-expression of CCR4 and CTLA-4 among uninfected subjects (Fig. 2A). In HTLV-1-infected subjects (AC and HAM/TSP), the levels of CCR4^+^ CTLA4^+^ CD4^+^ T cells among the CD39^+^ CD25^+^ CD4^+^ T cells significantly declined (p = 0.0159) compared to uninfected controls (Fig. 2B).

Increased PD-1 expression has been shown to mark T cell activation and dysfunction [66], [67], but its expression has also been used to discriminate Treg subsets [68]. We measured surface expression of PD-1 among the different CD4^+^ T cells and compared the expression among the three population groups according to CD25 and CD39 expression. We observed differential expression of PD-1 levels with CD39^+^CD25^+^CD4^+^ T cells (regulatory) having higher expression of PD-1 in HAM/TSP patients, (p = 0.0189) (Fig. S3 A,B), whereas the CD39^+^CD25^−^CD4^+^ T cells (inducer) significantly expressed lower levels of PD-1 in HAM/TSP infected patients compared to asymptomatic controls, (p = 0.0317) (Fig. S3 B). These data suggest that HTLV-1 infection alters the phenotypic repertoire of CD4 regulatory T cells to less anergic state with those patients with HAM/TSP and expand a population of CD39^+^CD25^−^ CD4^+^ T cells with lower PD-1 levels indicating a potential for even greater T-cell activity.

### Direct Association of CD39^+^CD25^−^CD4^+^ T Cells with HTLV-1 Proviral Load in HAM/TSP but not with AC Subjects

**Figure 3:**
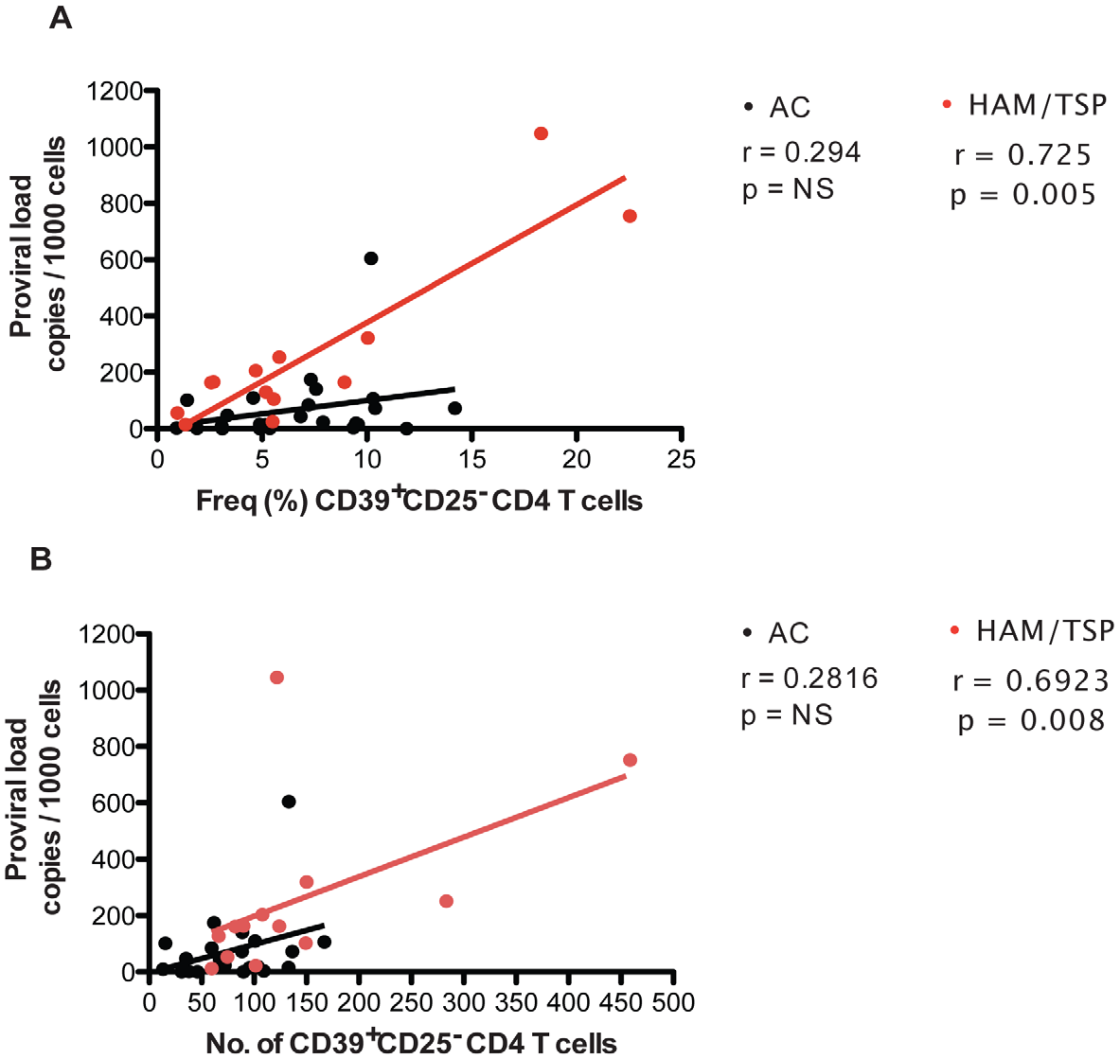
Association between HTLV-1 proviral load and CD39^+^CD25^−^CD4^+^ T cells in HAM/TSP patients. Frequency of CD39^+^CD25^−^ CD4^+^ T cells (A) and number of CD39^+^CD25^−^CD4^+^ T cells (B) were plotted against proviral load of AC and HAM/TSP patients. The statistical difference was deemed significant using a two-tailed Spearman test analysis if p<0.05.

High levels of HTLV-1 proviral load levels have been associated to variable Treg frequency as well as reduced HTLV-1-specific CD8^+^ T-cell lytic efficiency and frequency [14], [39], [54]. Because HTLV-1 infection promotes T-cell activation and proliferation [69] and proviral load levels are associated with CD4^+^ T-cell clonal expansion [70], we wished to assess if there is an association between proviral load and the CD39 expressing CD4^+^ T-cell subsets in 24 HTLV-1 asymptomatic carriers and 13 HAM/TSP patients. Frequency and number of CD39^+^CD25^−^CD4^+^ T cells from HAM/TSP patients but not from HTLV-1 asymptomatic carriers are associated with HTLV-1 proviral load levels (Fig. 3A,B). There was no association between proviral load and the frequency (Fig. S4A) or numbers (Fig. S4B) of CD39^+^CD25^+^ CD4^+^ T cells. Overall this data suggests that CD39^+^CD25^−^ CD4^+^ T cells may contribute to the increased rate of CD4^+^ T-cell proliferation and consequently higher levels of HTLV-1 proviral load in HAM/TSP patients.

### Reduction in IL-17 Production in HAM/TSP Patients

Th17 cells have a pivotal role in many autoimmune and inflammatory conditions, including Multiple Sclerosis (MS). Besides, CD39^+^ Tregs, a T-cell subset with increased frequency among HTLV-1-infected patients, suppress Th17 cells in healthy individuals but are dysfunctional in MS [49], a neurodegenerative disorder with some clinical similarities to HAM/TSP. Little is known about IL-17 production in HTLV-1 infection. It has been reported that expression of IL-17 mRNA is induced by Tax in HTLV-1 infected T-cell lines [43] and increased in periodontal tissue of HTLV-1-infected subjects with periodontitis [71]. However, IL-17 production from a specific CD4^+^ T-cell subset is reduced among HAM/TSP patients [44].

**Figure 4:**
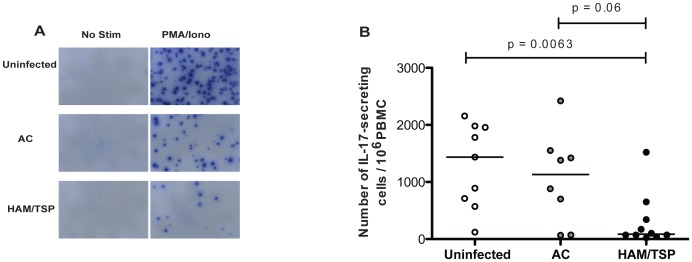
Measurement of IL-17 secretion of PBMC by ELISPOT. Experiments run in duplicate. Results are medium-subtracted and normalized to 10^6^ cells. The statistical difference was deemed significant using a Mann-Whitney U test analysis if p<0.05. Horizontal bars indicate median values. (A) ELISPOT data from 1 representative experiment of each group of subjects. (B) Number of IL-17 secreting cells in PBMC from 9 uninfected donors, 8 HTLV-1 asymptomatic carriers and 10 HAM/TSP patients.

We therefore assessed the proportion of IL-17 secreting cells using PBMCs from 18 HTLV-1 infected patients (8 asymptomatic carriers and 10 HAM/TSP) and 9 uninfected subjects stimulated with PMA and ionomycin in an ELISPOT assay. We found a significant smaller number of IL-17 secreting cells in HAM/TSP patients compared to uninfected subjects and a trend towards a reduced number of IL-17 secreting cells compared to HTLV-1 asymptomatic carriers (Fig. 4A, B).

We also found a significant smaller Th17/CD39^+^CD25^−^ T-cell ratio in HAM/TSP patients as compared to HTLV-1-infected asymptomatic carriers, considering the number of IL-17 secreting cells and the frequency or number of CD39^+^CD25^−^CD4^+^ T cells (Fig. S 5A). A similar reduction was found when we analyzed the Th17/CD39^+^CD25^+^ ratio in HAM/TSP patients and compared to HTLV-1-infected asymptomatic carriers, considering the number of IL-17 secreting cells and the frequency or the number of CD39^+^CD25^+^CD4^+^ T cells (Fig. S 5B).

### Increased IFN-γ, TNF-α ^+^IL-2^+^ Double Positive CD39^+^CD25^+^ CD4^+^ T Cells and Reduced IL-17 Expression in HAM/TSP Patients

**Figure 5:**
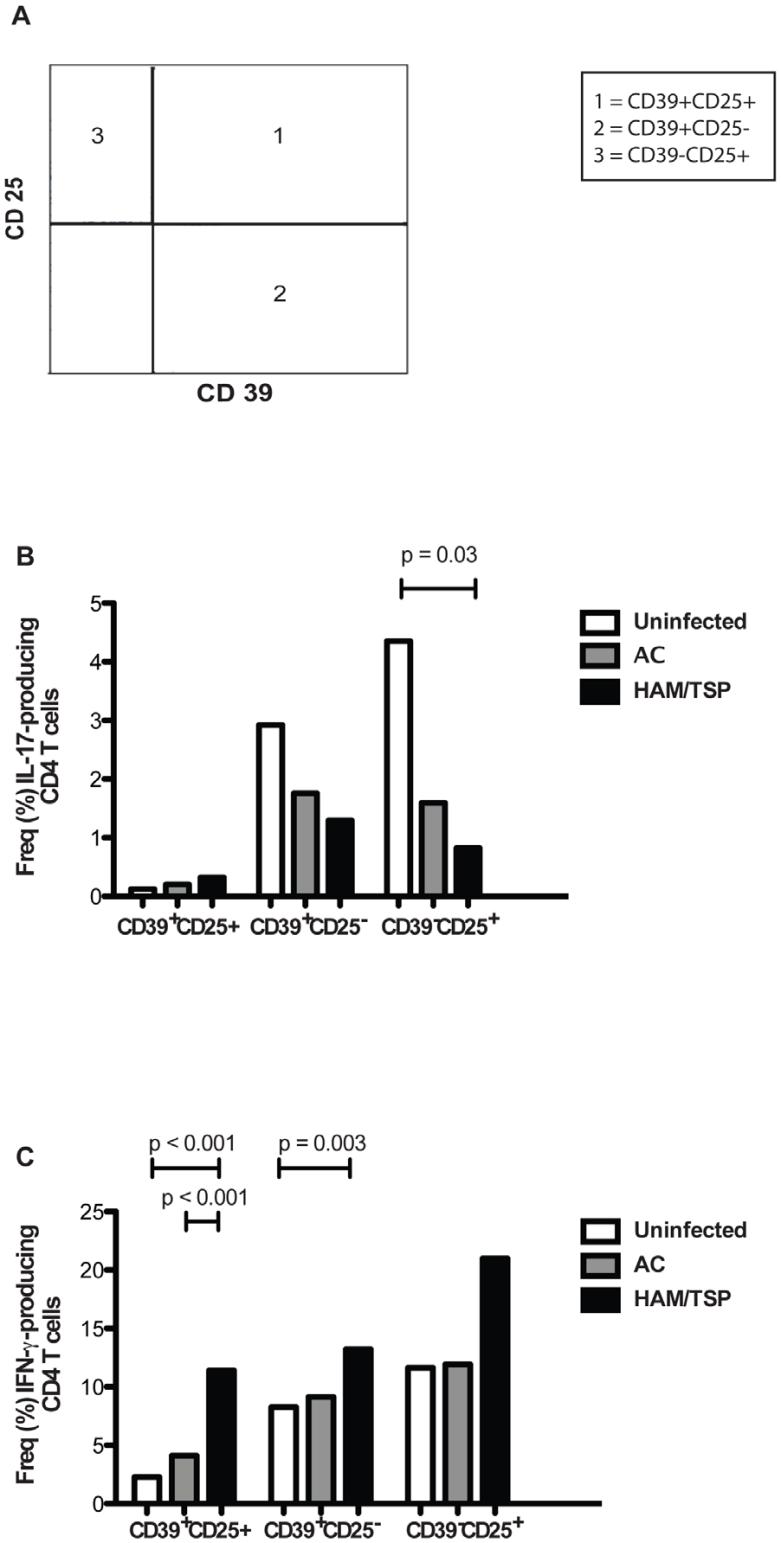
IL-17 and IFN-γ production by CD4^+^ T cells subsets. Subsets were numbered according to CD39 and CD25 expression (A): 1 = CD39^+^CD25^+^; 2 = CD39^+^CD25^−^; 3 = CD39^−^CD25^+^. Graph shows median of proportion of (B) IL-17-producing CD4^+^ T cells and (C) IFN-γ-producing CD4^+^ T cells subsets after PMA and ionomycin stimulation on PBMCs from 9 uninfected, 8 HTLV-1-infected asymptomatic carriers and 10 HAM/TSP patients. The statistical difference was deemed significant using a Mann-Whitney U test analysis if p<0.05.

HAM/TSP patients appears to have CD4^+^ T cells that are conditioned to produce IFN-γ [36]. Conflicting data regarding IL-17 production in HTLV-1 infected subjects and a possible role along with IFN-γ in the proinflammatory milieu observed in HAM/TSP patients led us to investigate the production of inflammatory cytokines of CD4^+^ T cells from 9 uninfected, 8 HTLV-1 asymptomatic carriers and 10 HAM/TSP patients. CD4^+^ T cells subsets from HAM/TSP subjects (CD39^−^CD25^+^CD4^+^ T cells) showed significant reduced IL-17 levels when compared to uninfected subjects (Fig. 5B), confirming our results from ELISPOT assays.

**Figure 6:**
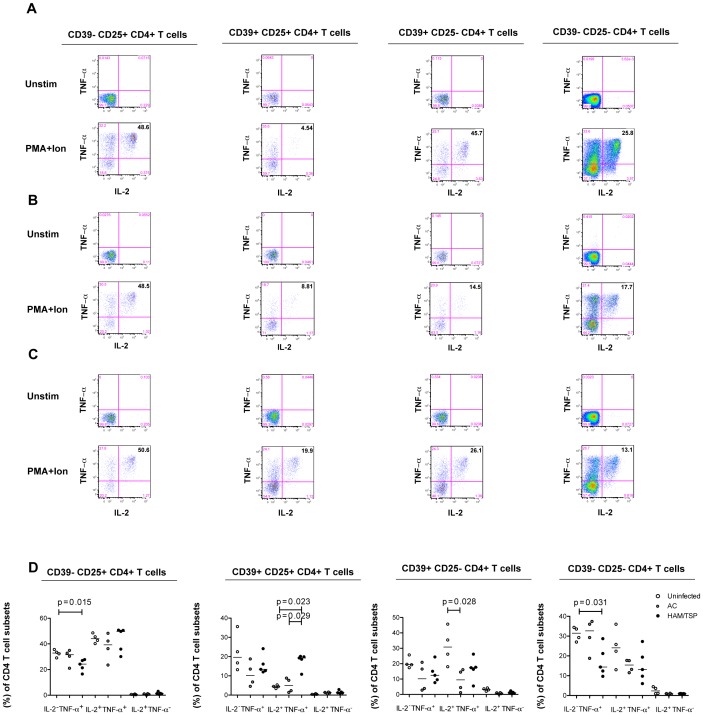
TNF-α and IL-2 production by CD4^+^ T cells based on CD39 and CD25 expression. The statistical difference was deemed significant using a Mann-Whitney U test analysis if p<0.05. Horizontal bars denote median values. TNF-α and IL-2 production by CD4^+^ T cells from (A) one representative uninfected donor, (B) one HTLV-1 asymptomatic carrier and (C) one HAM/TSP. (D) Production of TNF-α and IL-2 by CD4^+^ T cells in AC and HAM/TSP patients and uninfected donors based on CD39 and CD25 expression.

Also, we found a significant increased IFN-γ production among CD39^+^CD25^+^CD4^+^ T cells from HAM/TSP patients compared to HTLV-1 asymptomatic carriers and uninfected subjects (Fig. 5C). We also observed increased levels of IFN-γ in CD39^+^CD25^−^CD4^+^ T cells in HAM/TSP subjects compared to HTLV-1 seronegative individuals (Figure 5C). We next determined the proportion of TNF-α and IL-2 producing CD4^+^ T cells in uninfected subjects, HTLV-1 asymptomatic carriers and HAM/TSP patients (Fig. 6 A,B,C). Interestingly, we found a significant increased frequency of TNF-α^+^ IL-2^+^ producing cells in the CD39^+^CD25^+^ CD4^+^ T cells compartment among HAM/TSP patients compared to HTLV-1 asymptomatic carriers and uninfected subjects (Fig. 6D). This CD4^+^ T-cell subset has a suppressive phenotype and does not produce significant amounts of IFN-γ TNF-α and IL-2 in uninfected subjects, suggesting that these immunostimulatory and/or immunoregulatory CD39 expressing T cell subsets may participate in the pro-inflammatory milieu that could potentially lead to the progression to HAM/TSP.

### HBZ but not Tax Expression Associates with the Expanded CD39^+^CD25^−^CD4^+^ T-Cell Subset Seen in HAM/TSP

**Figure 7:**
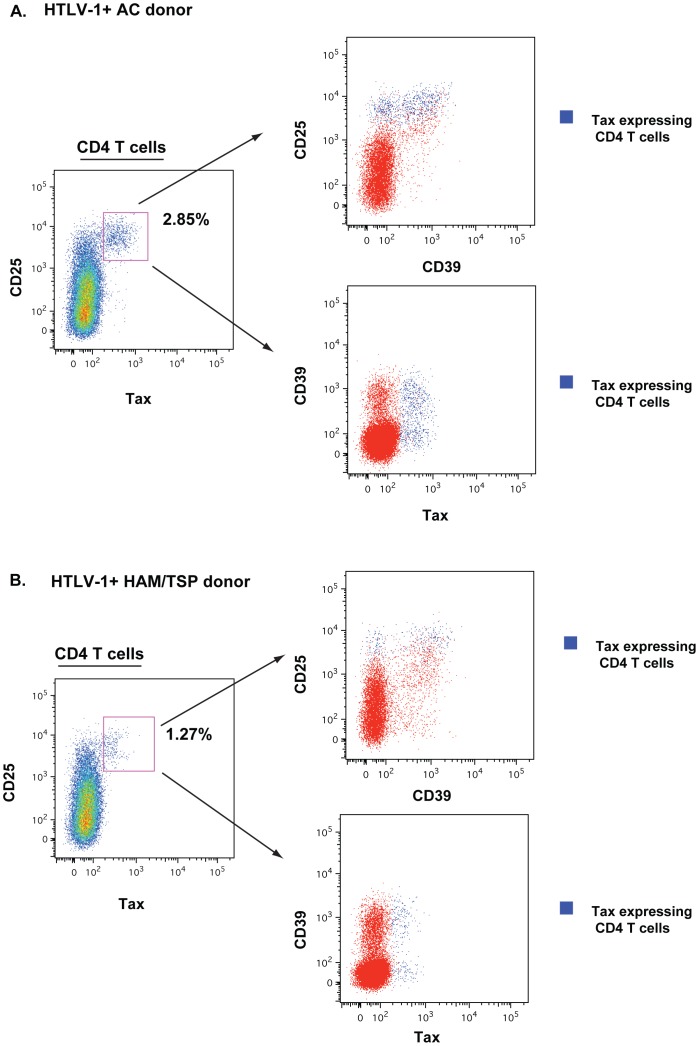
Tax expression in CD4^+^ T cells of HTLV-1-infected subjects. Representative flow cytometry data of CD25, CD39 and Tax expression in (A) one HTLV-1-infected asymptomatic carrier and (B) one HAM/TSP patient. Plots show Tax expression restricted to CD25^+^ CD4^+^ T cells regardless of CD39 expression.

**Figure 8:**
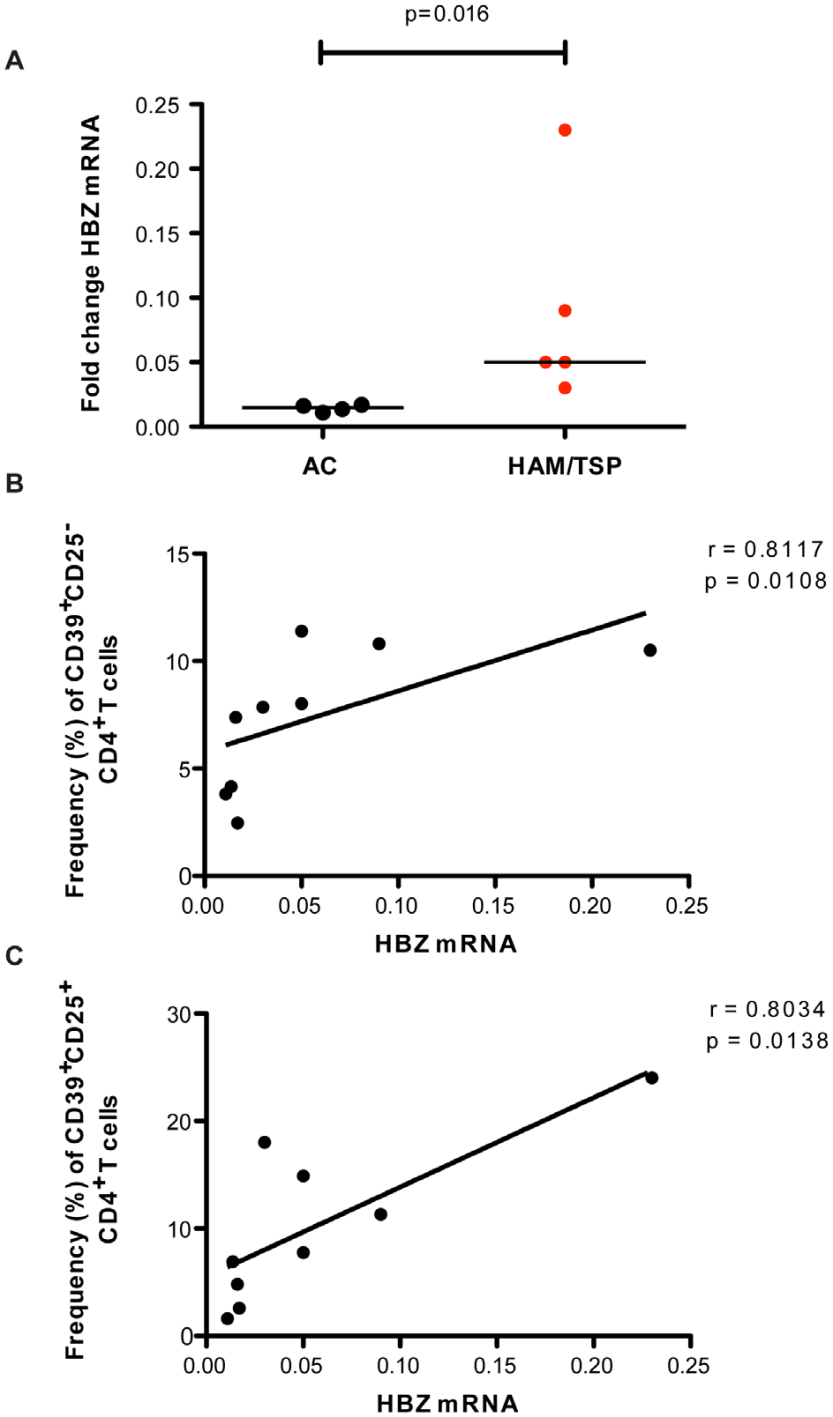
Relative HBZ mRNA expression. Expression was calculated as 2^−ΔΔCt^ using the mean of β-actin as housekeeping controls. The statistical difference was deemed significant using a Mann-Whitney U test analysis if p<0.05. Horizontal bars denote median values. (A) Fold change of HBZ mRNA expression in PBMCs of 4 ACs and 5 HAM/TSP patients. (B) Positive correlation between HBZ mRNA levels and frequencies of CD39^+^CD25^−^ CD4^+^ T cells in HTLV-1-infected patients. (C) Positive correlation between HBZ mRNA level and frequency of CD39^+^CD25^+^ CD4^+^ T cells in HTLV-1-infected patients.

The trans-acting viral regulatory protein Tax *(Tax)* gene and the HTLV-1 basic leucine zipper (*HBZ*) gene, an antisense transcribed mRNA promote proliferation and maintenance of HTLV-1 infected T cells and are reported to be oncogenic factors in ATL induction [25], [27], [72], [73]. Both have been identified as important targets implicated in the pathogenesis of HAM/TSP. To assay whether increased proviral load is responsible for the expansion of CD39^+^ CD4^+^ T-cell subsets, we evaluated protein Tax expression by flow and gene expression of *HBZ* and *Tax* as a proxy to determine HTLV-1 replication. HTLV-1-infected subjects were therefore assessed for Tax expression on the CD4^+^ T cells subsets based on CD39 and CD25 expression. We observed Tax expression was represented in the CD25 expressing CD4^+^ T cells subsets in both CD39 and CD39 lacking cells. There was no difference in the Tax expression representation between AC and HAM/TSP (Fig. 7A,B). HAM/TSP patients presented with a significantly higher level of HBZ mRNA compared to HTLV-1 asymptomatic carriers (Fig. 8A). This result is comparable to results previously reported by Saito *et al,* [27]. We assessed the relationship between *HBZ* transcription levels and the CD39 expressing immunoregulatory T cells. *HBZ* transcription levels positively associated with both CD39^+^CD25^+^ and CD39^+^CD25^−^CD4^+^ T cells (Fig. 8 B,C). These data suggest that Tax expression is absent among the CD39^+^CD25^−^CD4^+^ T cells that are expanded in HAM/TSP and may not be the driver for this expansion. Our findings further suggest that *HBZ* is an important viral protein that is associated with expansion of these immunoregulatory T cells populations as disease progresses.

## Discussion

In this study, we have demonstrated that CD39 serves as a marker of the profound phenotypic and functional changes in CD4^+^ T cells in HTLV-1 that potentially contribute to the development of HAM/TSP. Several important findings have been revealed from this work. First, an increased frequency of the immunostimulatory (CD39^+^CD25^−^) CD4^+^ T cells in HAM/TSP patients and its association with proviral load, a marker of disease status. These cells retain lower levels of PD-1 reflecting a phenotypic and functional shift in T-cell activity. Second, the reduced number of Th17 cells combined with conversion of CD4^+^ T cells with suppressive phenotype (CD39^+^CD25^+^) to those with increased IFN-γ, TNF-α and IL-2 production in patients with HAM/TSP. These data suggest a novel role for CD39 as a useful marker and potential mediator of the dysregulation of the CD4^+^ T-cell subsets in symptomatic HTLV-1 infection. Our interpretations lead us to conclude that these changes occur to either counterbalance the proinflammatory effect of HTLV-1 infection or directly participate in the immunopathology leading to HAM/TSP.

The high frequency of a T cell subset with immunostimulatory properties [48] in HAM/TSP patients, but not in asymptomatic carriers, suggests that CD39^+^CD25^−^ CD4^+^ T cells contribute to the increased CD4^+^ T-cell proliferation seen in HAM/TSP despite concomitant expansion of CD4^+^ T cells with suppressive phenotype (CD39^+^CD25^+^). In patients who develop HAM/TSP, such expansion may be insufficient to control the deleterious effect of the virus-induced inflammatory milieu. Whether HTLV-1 infection induces an increase or reduction in regulatory CD4^+^ T cells [39], [42], [54], [74], [75] remains unresolved. This may be attributable to the different phenotypic combination of markers used to define suppressive regulatory T cells. Defining and identifying suppressive T cells is a major challenge in the context of autoimmunity or infection. Activated CD4^+^ T cells may also express most markers used to define Treg including FoxP3, the forkhead transcription factor [76]. FoxP3 expression is subject to transient expression by T-cell activation and is localized in the nucleus [77], which limits functional analyses after intranuclear staining.

Initial studies with functional analysis of CD4^+^ CD25^+^ T cells suggested that Tregs are dysfunctional in HAM/TSP and Tax expression has direct inhibitory effect on FoxP3 expression and function [42], [54], [75], [78]. However, it has recently been demonstrated that an increased frequency of FoxP3 expression in CD4^+^ T cells in HTLV-1 infection persists. One possible mechanism is the high plasma levels of CC chemokine ligand 22 (CCL22) the CCR4 ligand, apparently inducing FoxP3 expression [74]. Our results support this latter hypothesis and that despite an increased frequency of FoxP3 or T-regulatory cells (CD25^+^CD4^+^), these cells transition from being anergic to displaying a polyfunctional cytokine feature and may contribute to the proinflammatory millieu leading to HAM/TSP.

Based on our findings, we propose that CD39 serves as a novel marker to delineate the phenotype and the role of suppressive CD4^+^ T cells and other CD4^+^ T-cell subsets in HAM/TSP patients. CD39^+^CD25^+^CD4^+^ T cells from HAM/TSP produce significantly higher levels of not only IFN-γ but also TNF-α and IL-2 and more importantly dual TNF-α^+^IL-2^+^ production within the CD39^+^CD25^+^ subset when compared to HTLV-1 asymptomatic carriers or uninfected subjects. It is likely that these cells may constitute a proportion of the T_HAM_ cells very elegantly described by Yamano *et al* [44]. Since expression of *HBZ* mRNA strongly correlates with the expansion of CD39^+^ expressing CD4^+^ T cells, HBZ may drive the shift of these T cells subsets to a polyfunctional status.

Tax expression and proviral load levels have been used as markers of disease progression [13], [79], but results with such markers are not consistent. Conversely, levels of HBZ mRNA were reported to strongly correlate with disease severity [27]. The positive correlation between HBZ mRNA levels and the frequency of both the CD39^+^CD25^+^ and CD39^+^CD25^−^ CD4^+^ T cell populations suggests that *HBZ* is associated with expansion of these immunoregulatory populations. Measuring CD39^+^CD25^−^CD4^+^ T cells frequency may be evaluated as a clinical index of disease progression to HAM/TSP as CD4^+^ T cells counts is used in HIV disease progression.

Several inflammatory cytokines have been implicated in the pathogenesis of HAM/TSP. IL-2, IFN-γ, and TNF-α levels are increased in HTLV-1 infection and contribute to neurological damage in HAM/TSP patients as well as in other neuroinflammatory conditions with clinical similarities to HAM/TSP such as Multiple Sclerosis [18], [21], [80], [81]. IL-17 also plays a pivotal role in the pathogenesis of HIV infection [50], [82] and various inflammatory diseases [83], but conflicting results regarding frequency of Th17 and IL-17 production were described in HTLV-1 infection [43], [44]. We predicted Th17 cells would be increased in HAM/TSP as in other inflammatory diseases along with the rise in T cells with suppressive phenotype to suppress the inflammatory process. Surprisingly, the reduced number of Th17 cells in PBMC from HAM/TSP patients combined with the increased frequency of CD4^+^ T cells with suppressive phenotype (CD39^+^CD25^+^) resembles results of our previous studies and from others in HIV-1 infection [50], [82], [84], where we demonstrate that IL-2 mediates expansion of CD4^+^ T cells expressing CD25, FoxP3, and lacking CD127, with a negative effect over Th17 cells frequency. Lower frequency of Th17 cells combined with high frequency of CD4^+^ T cells with suppressive phenotype (CD39^+^CD25^+^) results in a reduced ratio of Th17 cells and CD39^+^CD25^+^CD4^+^ T cells in HAM/TSP. Thus, the polarization of CD4^+^ T-cell responses towards Th1 in HAM/TSP [85] may be the result or cause the hitherto imbalance of Th17 and suppressive CD4^+^ T cells seen in these patients. A thorough analysis of Th17 cells in the CNS of HAM/TSP patients is warranted to clarify whether this Th17 reduction is restricted to peripheral blood or if these alterations are seen in sites where the inflammatory process have clinical impact.

Changes in frequency of CD39 expressing CD4^+^ T cells may be an important component of the alterations seen in CD4^+^ T-cell responses to HTLV-1 infection. Increased frequency of CD4^+^ T cells with immunostimulatory properties may be one of the missing parts to understand the development of HAM/TSP despite of increased frequency of CD4^+^ T cells with suppressive phenotype in this condition. To determine the frequency of CD39^+^CD25^−^CD4^+^ T cells in HTLV-1 infection should be considered as a proxy to disease progression. Besides, reduced levels of IL-17 and increased IFN-γ, IL-2 and TNF-α confirms the skewed Th1 specificity of HTLV-1-related inflammatory alterations and immunotherapy to restore Th17 cells may be a tool to downregulate Th1 responses. Finding these changes in inflammatory sites such as the CNS will determine if the prevention or reversion of these alterations may represent a valuable goal for modifying HAM/TSP clinical course.

## Supporting Information

Figure S1 **Dot plots of CD25, CD127 and FoxP3 expression on CD4^+^ T cells.** (A) FoxP3 expression in CD25^hi^ CD127^low^ CD4^+^ T cells. (B) Increased proportion of CD25^+^FoxP3^+^CD127^low^ CD4^+^ T cells in HTLV-1-asymptomatic carriers and HAM/TSP patients compared to uninfected subjects.
(TIF)

Figure S2 **(A) Number of total CD4^+^ T cells in uninfected donors, HTLV-1-asymptomatic carriers and HAM/TSP patients.** (B) Proportion and (C) number of CD39^−^CD25^+^ CD4^+^ T cells in uninfected donors, HTLV-1-asymptomatic carriers and HAM/TSP patients.
(TIF)

Figure S3 **Expression of PD-1 on CD4 T cells of uninfected subjects, HTLV-1 asymptomatic carriers and HAM/TSP patients based on CD39 and CD25 expression.** The statistical difference was deemed significant using a Mann-Whitney U test analysis if p<0.05. * indicates p<0.05. Horizontal bars denote median values. (A) PD-1 expression on CD4^+^ T cells from one representative uninfected donor, one HTLV-1-infected-asymptomatic carrier and one HAM/TSP patient. (B) Proportion of expression of PD-1 in CD39^+^CD25^+^ and CD39^+^CD25^−^ CD4^+^ T cells of uninfected donors, AC and HAM/TSP patients.
(TIF)

Figure S4 **Correlation between HTLV-1 proviral load and frequency and number of CD39^+^CD25^+^ CD4^+^ T cells in HTLV-1-asymptomatic carriers and HAM/TSP patients.** (A) Frequency of CD39^+^CD25^+^ CD4^+^ T cells and (B) number of CD39^+^CD25^+^ CD4^+^ T cells were plotted against proviral load of AC and HAM/TSP patients.
(TIF)

Figure S5 **IL-17 production by the different subsets of CD4^+^ T cells.** (A) Th17/Tind cells ratio from number of IL-17 producing cells and frequency and number of CD39^+^CD25^−^ CD4^+^ T cells of 10 HAM/TSP patients, 8 HTLV-1 asymptomatic carriers and 9 uninfected donors. Horizontal bars indicate mean values. (B) Th17/Treg cells ratio from number of IL-17 secreting cells and frequency and number of CD39^+^CD25^+^CD4^+^ T cells of 10 HAM/TSP patients, 8 HTLV-1 asymptomatic carriers and 9 uninfected donors. The statistical differences were deemed significant using a Mann-Whitney U test analysis if p<0.05. Horizontal bars indicate mean values.
(TIF)
