# Supplementary figures and images for: Expansion in CD39+ CD4+ Immunoregulatory T Cells and Rarity of Th17 Cells in HTLV-1 Infected Patients Is Associated with Neurological Complications
Source: PLoS Negl Trop Dis. 2013 Feb 7;7(2):e2028. doi: 10.1371/journal.pntd.0002028 (PMC3566991; doi:10.1371/journal.pntd.0002028)

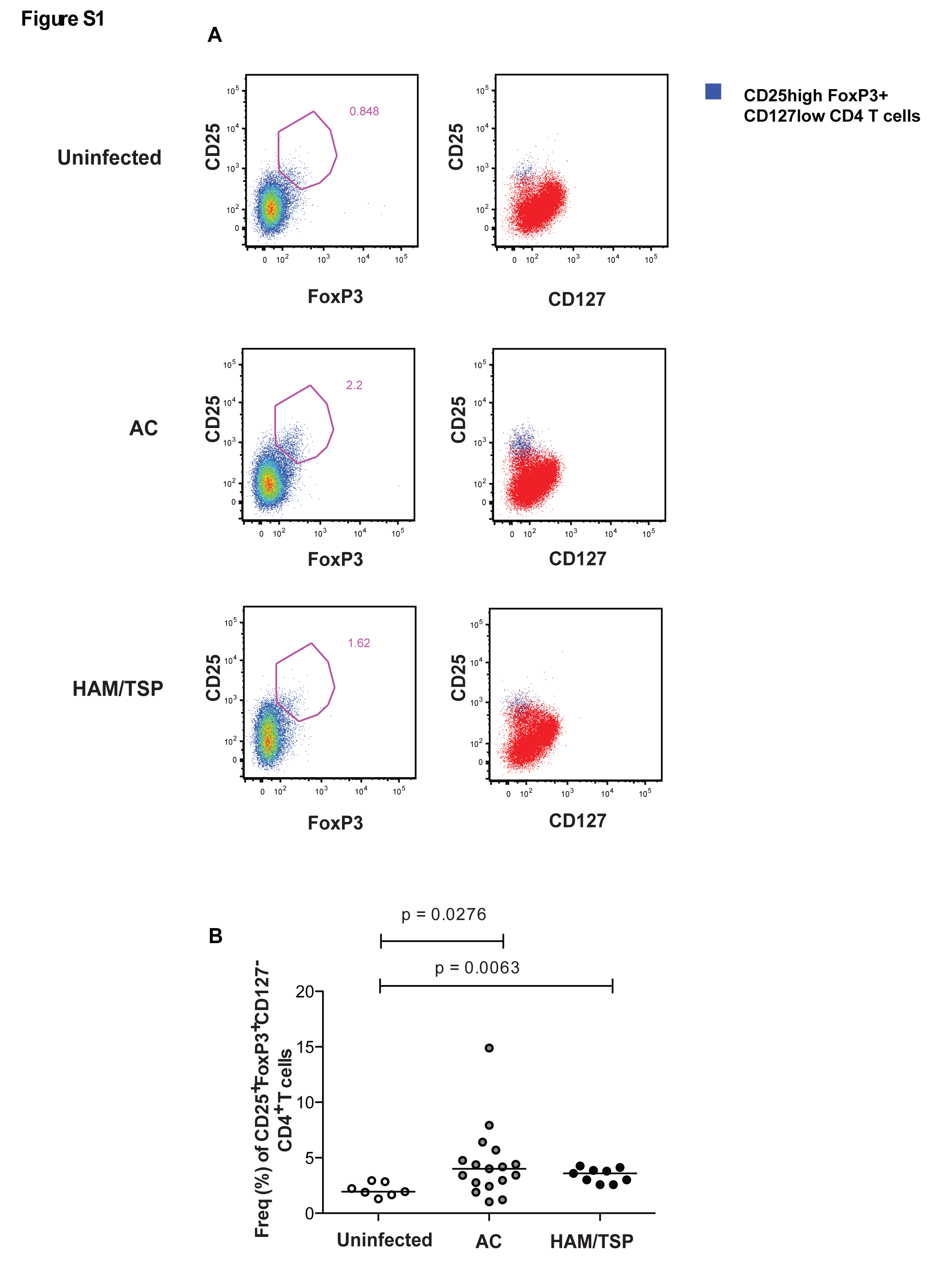

Supplement: Figure S1 — Dot plots of CD25, CD127 and FoxP3 expression on CD4+ T cells. (A) FoxP3 expression in CD25hi CD127low CD4+ T cells. (B) Increased proportion of CD25+FoxP3+CD127low CD4+ T cells in HTLV-1-asymptomatic carriers and HAM/TSP patients compared to uninfected subjects. (TIF) [file pntd.0002028.s001.tif]

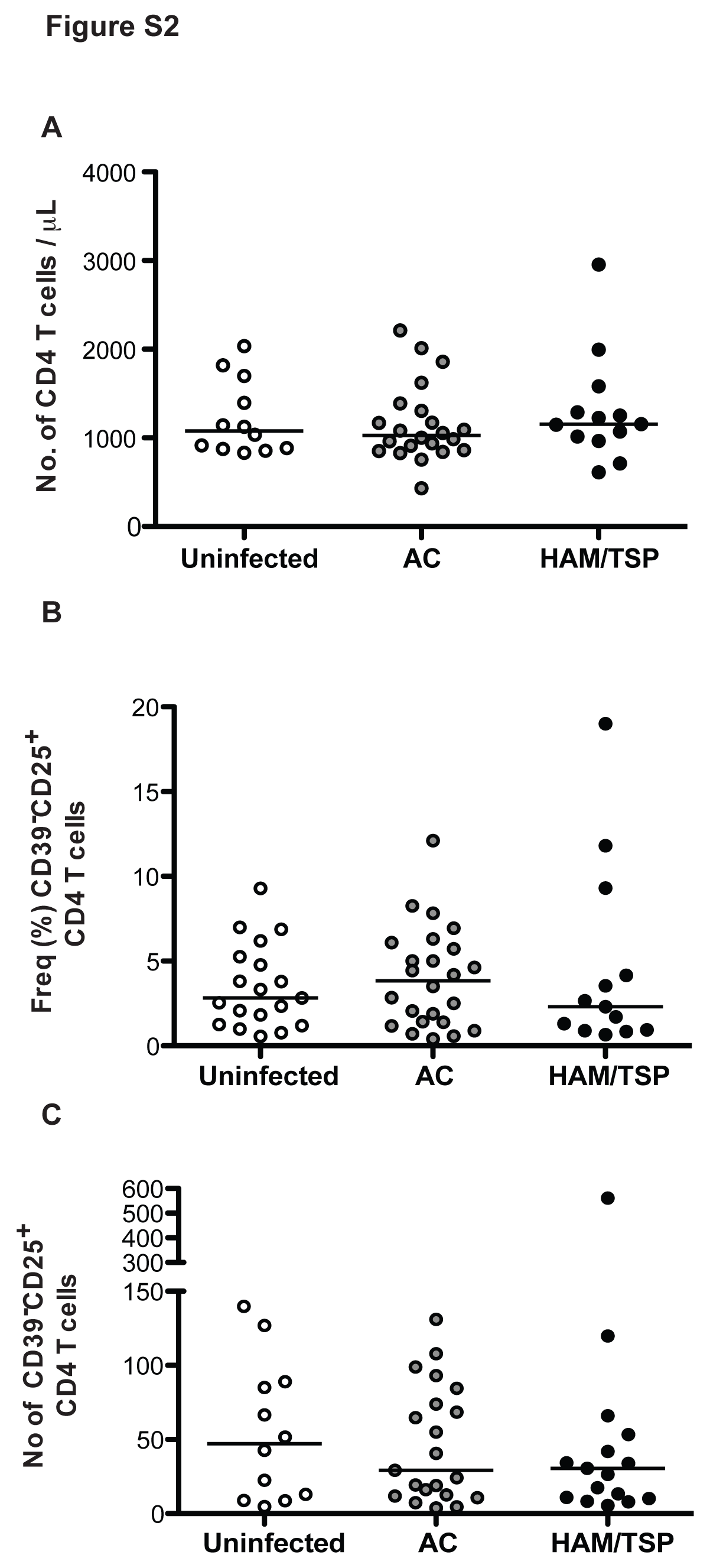

Supplement: Figure S2 — (A) Number of total CD4+ T cells in uninfected donors, HTLV-1-asymptomatic carriers and HAM/TSP patients. (B) Proportion and (C) number of CD39−CD25+ CD4+ T cells in uninfected donors, HTLV-1-asymptomatic carriers and HAM/TSP patients. (TIF) [file pntd.0002028.s002.tif]

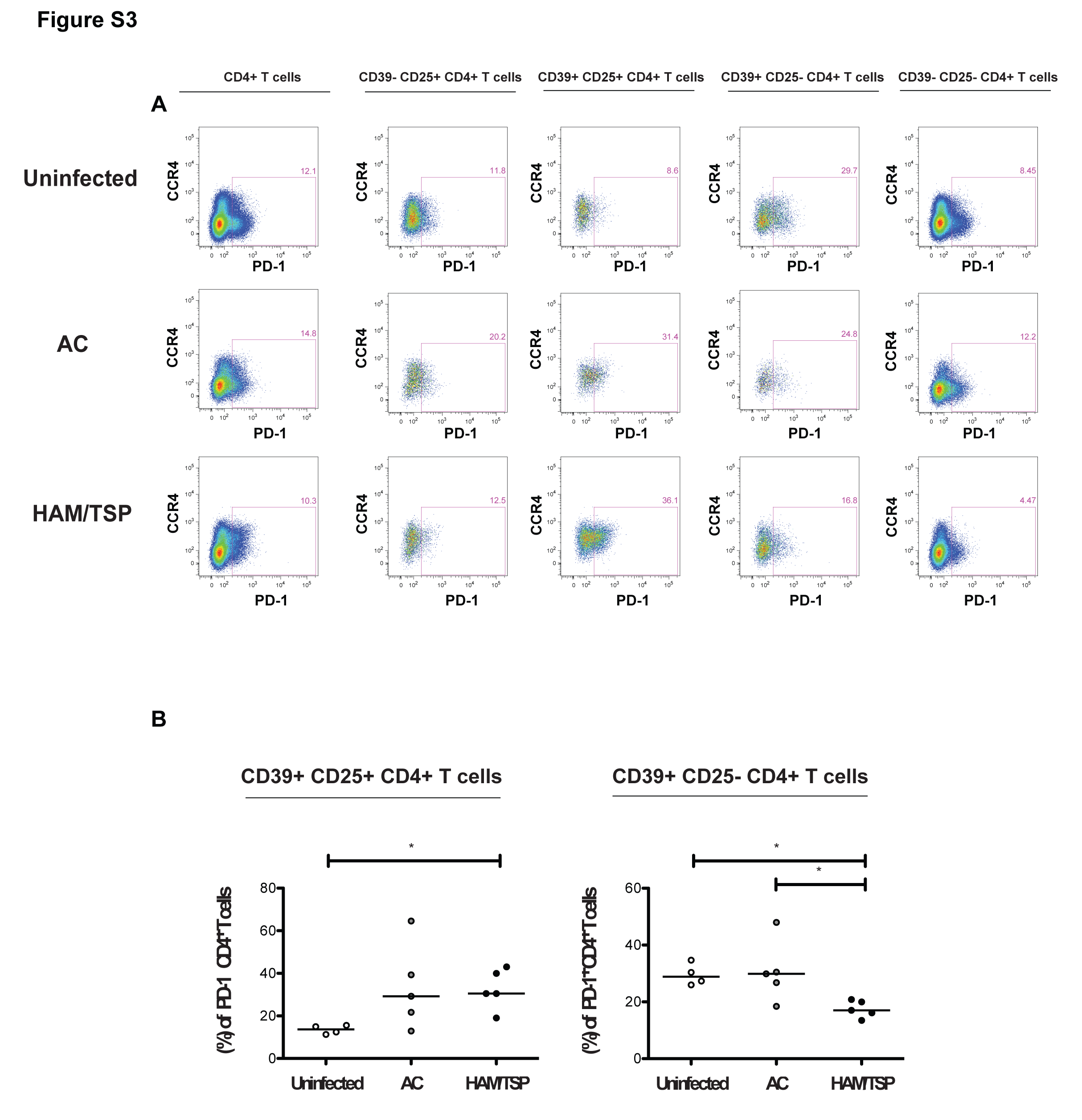

Supplement: Figure S3 — Expression of PD-1 on CD4 T cells of uninfected subjects, HTLV-1 asymptomatic carriers and HAM/TSP patients based on CD39 and CD25 expression. The statistical difference was deemed significant using a Mann-Whitney U test analysis if p<0.05. * indicates p<0.05. Horizontal bars denote median values. (A) PD-1 expression on CD4+ T cells from one representative uninfected donor, one HTLV-1-infected-asymptomatic carrier and one HAM/TSP patient. (B) Proportion of expression of PD-1 in CD39+CD25+ and CD39+CD25− CD4+ T cells of uninfected donors, AC and HAM/TSP patients. (TIF) [file pntd.0002028.s003.tif]

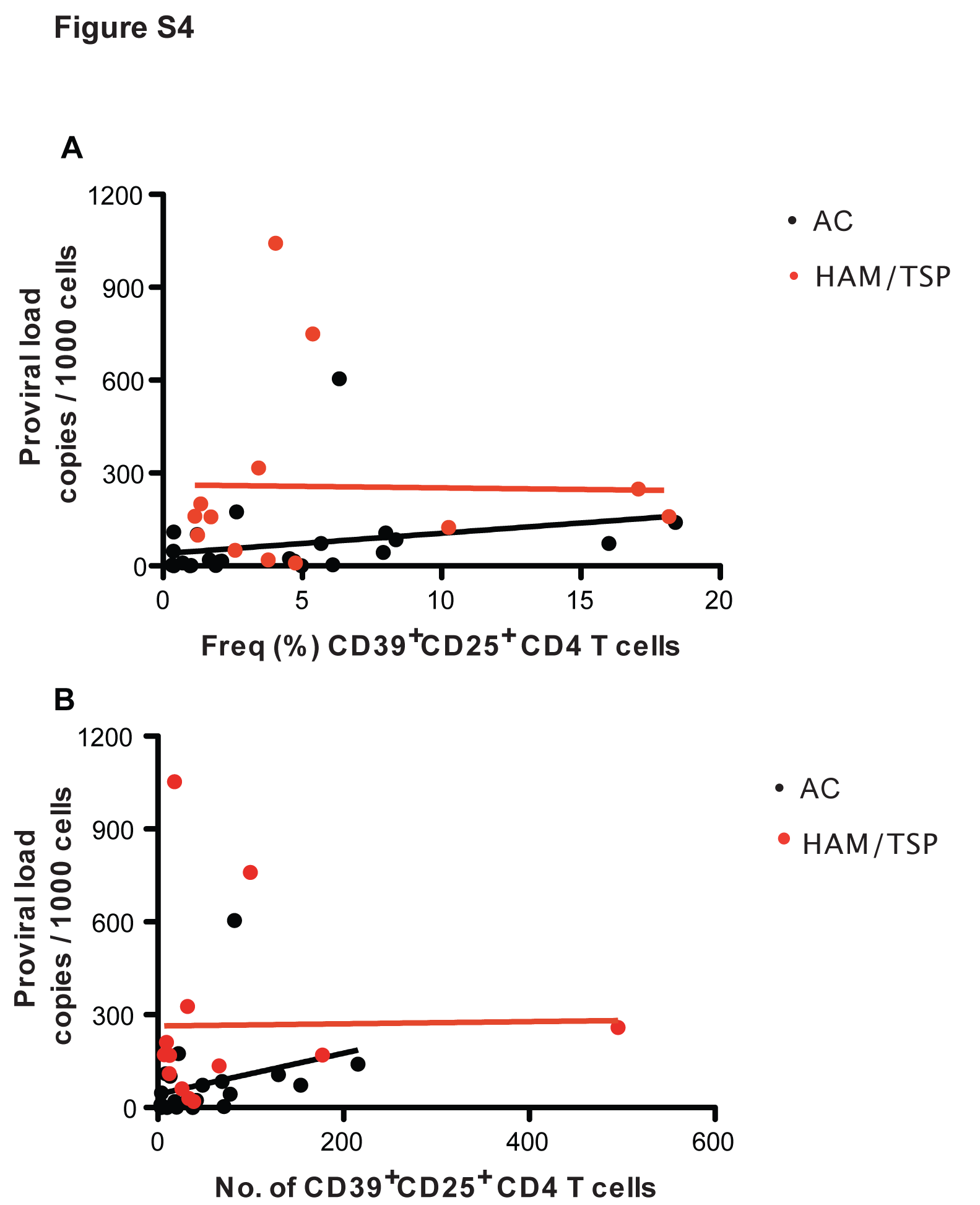

Supplement: Figure S4 — Correlation between HTLV-1 proviral load and frequency and number of CD39+CD25+ CD4+ T cells in HTLV-1-asymptomatic carriers and HAM/TSP patients. (A) Frequency of CD39+CD25+ CD4+ T cells and (B) number of CD39+CD25+ CD4+ T cells were plotted against proviral load of AC and HAM/TSP patients. (TIF) [file pntd.0002028.s004.tif]

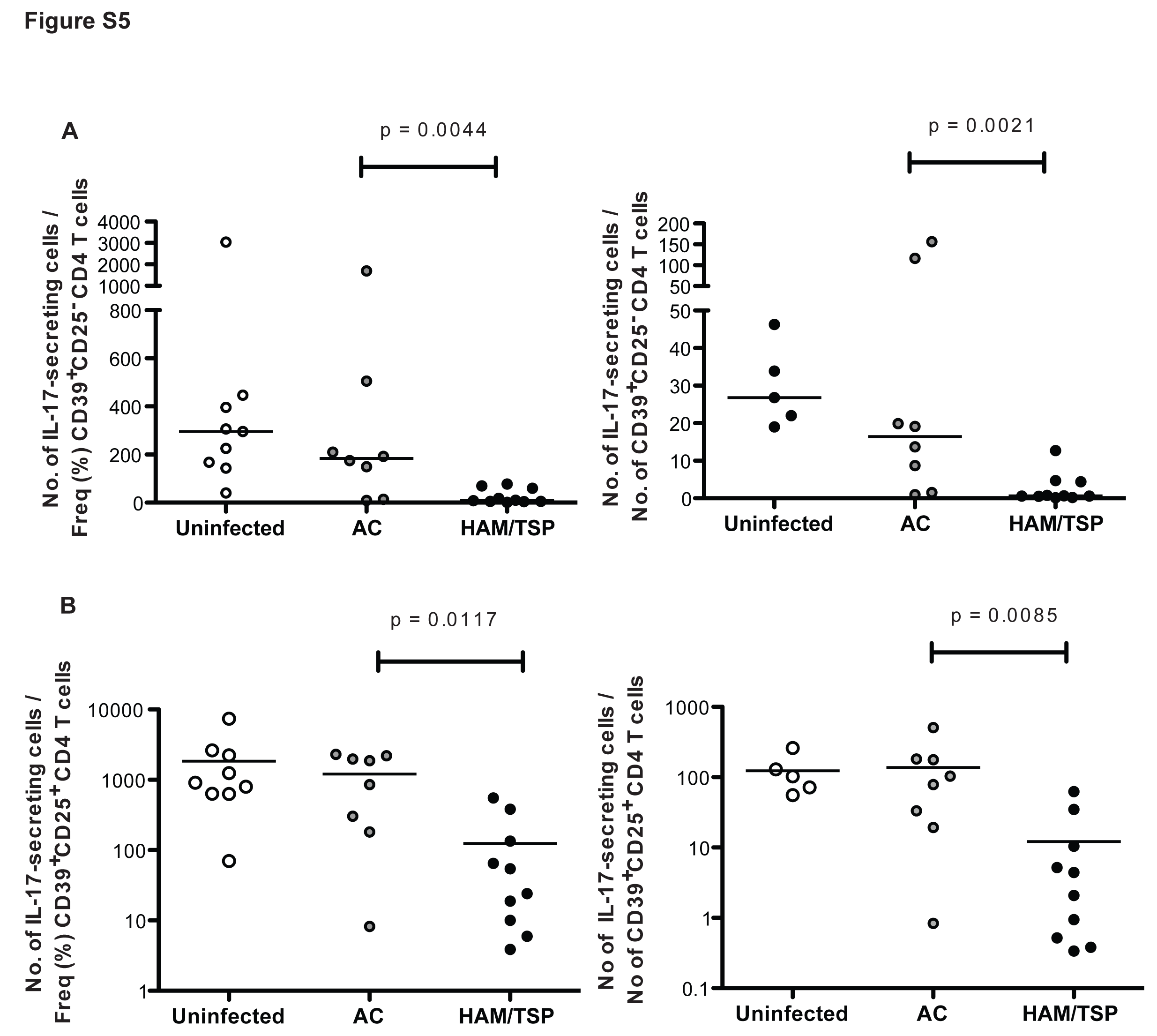

Supplement: Figure S5 — IL-17 production by the different subsets of CD4+ T cells. (A) Th17/Tind cells ratio from number of IL-17 producing cells and frequency and number of CD39+CD25− CD4+ T cells of 10 HAM/TSP patients, 8 HTLV-1 asymptomatic carriers and 9 uninfected donors. Horizontal bars indicate mean values. (B) Th17/Treg cells ratio from number of IL-17 secreting cells and frequency and number of CD39+CD25+CD4+ T cells of 10 HAM/TSP patients, 8 HTLV-1 asymptomatic carriers and 9 uninfected donors. The statistical differences were deemed significant using a Mann-Whitney U test analysis if p<0.05. Horizontal bars indicate mean values. (TIF) [file pntd.0002028.s005.tif]
